# Association of Mitral Annulus Calcification with High-Sensitivity C-Reactive Protein, Which Is a Marker of Inflammation

**DOI:** 10.1155/2012/606207

**Published:** 2012-02-22

**Authors:** Ertuğrul Kurtoğlu, Hasan Korkmaz, Erdal Aktürk, Mücahid Yılmaz, Yakup Altaş, Ahmet Uçkan

**Affiliations:** ^1^Department of Cardiology, Elazığ Education and Research Hospital, 23100 Elazığ, Turkey; ^2^Department of Cardiology, Adıyaman University Faculty of Medicine, 02040 Adıyaman, Turkey; ^3^Department of Cardiology, Sevgi Hospital, 44040 Malatya, Turkey

## Abstract

*Objectives*. There are limited clinical data revealing the relationship between mitral annular calcification (MAC) and systemic inflammation. The goal of the present study was to compare high-sensitivity C-reactive protein (hs-CRP) levels in patients with and without MAC and investigate the relationship between MAC and hs-CRP. *Methods*. One hundred patients with MAC who underwent transthoracic echocardiography (TTE) and 100 age-matched controls without MAC who underwent TTE were included in our study. Hs-CRP levels were compared between groups. *Results*. Prevalence of female gender, hypertension, and coronary artery disease were significantly higher in the MAC group than in the control group (64% versus 45%, *P* = 0.007, 42% versus 28%, *P* = 0.03 and 37% versus 18%, *P* = 0.003, resp.). On multivariate analysis, age, gender, and coronary artery disease were the only independent predictors of MAC. The levels of hs-CRP were higher in the MAC group than in the control group (2.02 ± 0.35 versus 1.43 ± 0.47 mg/dl, *P* < 0.001). This increase in hs-CRP levels in the MAC group persisted in patients without hypertension, coronary artery disease, and in male patients when compared to the control group. *Conclusions*. Our study demonstrated that hs-CRP, which is a sensitive marker of systemic inflammation, increased in patients with MAC.

## 1. Introduction

Mitral annular calcification (MAC) represents a chronic and degenerative calcification of the surrounding fibrous support of the mitral valve. This process is a common finding on echocardiographic examination and progresses with advanced age [1]. Although the exact mechanism is not well understood, MAC appears to be associated with cardiovascular risk factors and clinical atherosclerosis, thus giving additional evidence for the hypothesis that annular calcification is a form of atherosclerosis [2–4].With the recognition that systemic inflammation plays an important role in the development of atherosclerosis and has an important etiologic factor of cardiovascular disease [5], it is possible that this condition may also play a role in the development or progression of MAC. There are limited clinical data supporting the association between MAC and systemic inflammation. High-sensitivity C-reactive protein (hs-CRP) is a well-known and sensitive marker for prediction of the degree of systemic inflammation [6–10]. The purpose of our study was to compare the levels of hs-CRP in patients with MAC and those without MAC.

## 2. Materials and Methods

### 2.1. Study Population

Participants were recruited from patients admitted to cardiology department of our hospital. One hundred consecutive patients in whom a diagnosis of MAC was made by transthoracic echocardiography (MAC group) and 100 consecutive age-matched patients without MAC (control group) were included in our cross-sectional study. Patients were excluded if they had rheumatic heart disease, congestive heart failure, cardiomyopathy, prosthetic valves, renal failure (estimated glomerular filtration ratio (eGFR) below 90 mL/min/1.73 m^2^ or serum creatinine >1.0 mg/dL [11]), active inflammatory or infective disease, hematologic disorder, and malignant diseases. The study was approved by the local ethical committee and written informed consent was obtained from all patients.

### 2.2. Echocardiographic Technique

Complete 2-dimensional transthoracic and doppler colour flow measurements were performed in all patients with the 4-MHz transducer of a Phillips ATL 5000 HD (Bothell, WA, USA). The echocardiographic diagnosis of MAC was made by 2-dimensional and M-mode transthoracic echocardiography. On 2-dimensional echocardiography, the diagnosis included an intense echo-producing structure located at the junction of the atrioventricular groove and posterior mitral valve leaflet on the parasternal long-axis, apical 4-chamber, or parasternal short-axis views [12]. On M-mode echocardiography, the diagnosis depended on the appearance of an echocardiographically dense band throughout systole and diastole, and located behind the posterior leaflet and anterior to the left ventricular posterior wall [13]. Two experienced echocardiologist unaware of the patients' clinical data performed all echocardiographic evaluations. A patient was chosen for the study only when the 2 echocardiologists were in agreement about the absence or presence of MAC. Subjects were excluded if there was disagreement between two echocardiologists.

### 2.3. Clinical Data

All patients' demographic parameters were recorded and cardiac risk factors were determined: coronary artery disease (CAD) (>30% luminal diameter narrowing of ≥1 coronary artery by angiography [14], history of coronary revascularization, an abnormal myocardial perfusion scan or dobutamine stress echocardiogram, regional left ventricular akinesia and/or dyskinesia on echocardiogram, or pathologic Q waves on 12-lead electrocardiogram), systemic hypertension (blood pressure >140/90 mm Hg or ongoing antihypertensive medication), diabetes mellitus (fasting serum glucose level >126 mg/dL or ongoing diabetes medication), hypercholesterolemia (fasting serum total cholesterol level >200 mg/dL or ongoing lipid-lowering therapy), past or present tobacco use.

### 2.4. High-Sensitivity C-Reactive Protein

A 3 mL sample of peripheral venous blood was obtained from all patients after echocardiography for the measurement of hs-CRP and studied daily. Hs-CRP was measured using Cobas Iutegra 700 Analyzer (Roche Diagnostics GmbH, Mannheim, Germany).

### 2.5. Statistical Analysis

Statistical analyses were performed with the use of SPSS software, version 17.0 (SPSS). Continuous variables were presented as means ± SD and categorical variables as percentages. Differences between study groups in baseline characteristics were assessed with the use of two-sided Fisher's exact tests and chi-square tests for categorical variables and Student's *t* tests for continuous variables. Multivariate binary logistic regression analysis was performed to determine which clinical variables would independently predict the development of MAC. MAC entered into model as a dependent variable. Results were presented as odds ratios and 95% confidence intervals. Multiple linear regression analysis was used to examine the effect of clinical variables on hs-CRP levels. Hs-CRP level entered into model as a dependent variable (Table 4). Results were presented as beta coefficients and 95% confidence intervals. All *P* values were two-tailed, and values of less than 0.05 were considered to indicate statistical significance.

## 3. Results

Baseline characteristics and clinical data of 100 patients with MAC were compared with baseline characteristics and clinical data of 100 age-matched patients without MAC and results were summarized in Table 1. There were more women in the MAC group than in the control group. Hypertension and coronary artery disease were more frequent in the MAC group than in the control group. There were no statistically significant intergroup differences between MAC and control groups in diabetes mellitus, hypercholesterolemia, cigarette smoking, and medication use (Table 1). Multivariate logistic regression analysis identified age (odds ratio (OR) 1.09, *P* = 0.01), female gender (OR 2.06, *P* = 0.02) and coronary artery disease (OR 0.45, *P* = 0.02) as the independent variables significantly associated with MAC (Table 2). The adjusted R^2^ value was 0.45 for this model.

Serum hs-CRP levels in the MAC and control groups were summarized in Table 3. Significant differences in serum hs-CRP concentrations were found between patients with MAC and those without MAC. The levels of hs-CRP in both female and male patients with MAC were also significantly higher than those without MAC. Patients with CAD in the MAC group had higher hs-CRP levels than those with CAD in the control group. There was also statistically significant differences in hs-CRP levels between patients without CAD in the MAC and control group. Any statistically significant difference was not found between hypertensive patients with and without MAC, though the first group had a higher plasma hs-CRP level. Inversely, patients without hypertension in the MAC group had higher hs-CRP levels than those patients without hypertension in the control group. In multiple linear regression analysis, gender (*β* = 0.26, *P* < 0.001), hypertension (*β* = 0.19, *P* = 0.002), coronary artery disease (*β* = 0.22, *P* < 0.001), hypercholesterolemia (*β* = 0.18, *P* = 0.003), and smoking (*β* = 0.15, *P* = 0.016) were all independently associated with the levels of hs-CRP. The adjusted R^2^ value was 0.38 for this model.

## 4. Discussion

Mitral annular calcification (MAC) is a chronic process involving fibrosis and calcification of the mitral valve support ring and commonly observed on echocardiograms. Generally, this process is regarded benign although it is thought to produce significant regurgitation and/or stenosis in rare circumstances [15]. Consistent with studies describing the contributions of inflammation to atheroma formation [5, 16] and valve calcification [17], interest in inflammatory markers has increased because of their potential role as prognostic markers. Our study aimed to search this issue. Hs-CRP was used in this study as it is the most frequently used sensitive marker in systemic inflammation. In our study, mean hs-CRP levels were significantly lower in the control group compared with those in the MAC group indicating that a systemic inflammatory response was present in patients with MAC.

Although a number of hypotheses have been proposed to elucidate the ectopic deposition of calcium on cardiac structures [18, 19], the weight of evidence points toward a strong relationship between the calcification of the fibrous skeleton of the base of heart (mitral and aortic annuli) and vascular atherosclerotic changes. Experimentally induced systemic arterial atherosclerosis is associated with the deposition of fatty plaques on the aortic surface of the aortic valve cusps and the ventricular surface of the posterior mitral leaflet [20]. As the fatty plaques grow, their nutritional needs fail to be met, and they degenerate into calcific deposits. This finding was supported by pathological study showing that collections of foam cells may be observed on the endothelium of the epicardial coronary arteries, on the ventricular surface of the posterior mitral leaflet and on the aortic aspects of each of the aortic valve cusps and already found in adolescence and the second and third decades of life [21]. These data suggest that the presence of MAC probably indicates a systemic atherosclerotic process which involves aortic valve, mitral valve, coronary arteries, and perhaps other parts of the arterial systems. Thus, MAC may serve as a marker of subclinical atherosclerotic disease. In our study, CAD was more prevalent in the MAC group and this finding supports this theory. In addition to the abovementioned mechanisms, several studies have shown that MAC in elderly women but not in men can be attributed to ectopic calcium deposits, related to the severe bone loss caused by postmenopausal osteoporosis [22–24] and hypovitaminosis D [25].

When the literature is reviewed, MAC is observed more in patients who have atherosclerotic risk factors such as CAD and HT [2, 12, 26]. In addition, CAD and HT increase hs-CRP levels [9, 27]. In our study, patients with and without CAD in the MAC group were higher hs-CRP levels than those with and without CAD in the control group. But interestingly, patients without HT in the MAC group had higher hs-CRP levels than those without HT in the control group, which may indicate the presence of inflammation in patients with MAC. Also, compared with hypertensive patients in the MAC group, nonhypertensive patients in the same group had slightly, but not significantly, higher hs-CRP levels. This may be due to advanced stage of MAC in this group of patients.

It is well known that MAC is more common in females [28]. In our study, the proportion of female gender in the MAC group was significantly higher than the control group and this finding do not differ from most previous clinical studies [28–30]. This gender predominance in the prevalence of MAC seems paradoxical when taking into account the male predominance in prevalence of atherosclerosis, coronary artery disease, and calcific deposits in the coronary tree [28]. In our opinion, an explanation of this paradox may be grounded in possible different pathogenesis of MAC between women and men. In addition, when hs-CRP levels were compared by gender, males and females with MAC had higher levels than the ones in the control group who have the same gender. Also, our logistic regression analysis showed that gender was an independent risk factor for MAC.

The association of coronary artery disease and hypertension with MAC, as noted in the present study, is consistent with the findings of previous studies [31–33]. In addition, our logistic regression analysis indicated that older age and coronary artery disease but not hypertension were independent risk factors for MAC. On the other hand, there was no significant relationship between MAC and other traditional risk factors for cardiovascular disease including hypercholesterolemia, smoking status, and diabetes. Various studies have shown inconsistent relationships between atherosclerotic risk factors and MAC. For example, Nair et al. looked at the clinical characteristics of patients with MAC and found that, though there was a significant association between MAC and both diabetes and systemic hypertension, there was no significant relationship between MAC and serum cholesterol [34]. Adler et al. examined MAC patients undergoing carotid duplex ultrasound and found that, though MAC was associated with atherosclerotic disease, it was not associated with smoking or gender [35]. Allison et al. found that individuals with MAC were more likely to be older and have a history of hypertension and smoking, but diabetes and hyperlipidemia were not associated with MAC [36]. These differences with our study might be explained in part by patient population and/or statistical power.

Findings of our study are partly consistent with the results of the Cardiovascular Health Study which found an association between MAC and hs-CRP levels [37]. However, in the Cardiovascular Health Study, prevalent CAD and all cardiovascular risk factors including hypertension, diabetes, smoking, and hypercholesterolemia were significantly higher in the MAC group. All these factors may have contributed to increase hs-CRP level. In that study, the relationship between MAC and hs-CRP has not been examined in detail even if it revealed the relationship between them. Our study aims to demonstrate the contributions of these risk factors to the relationship between MAC and hs-CRP. High levels of hs-CRP in our MAC group show an association between MAC and hs-CRP. However, our study is not explanatory enough in terms of cause and effect relationship. This study does not clarify whether MAC itself increases hs-CRP or MAC occurs because CRP increases or there are other factors affecting these two situations. However, our logistic regression analysis identified age, female gender, and CAD as independent risk factors for MAC, whereas HT was not, which may be explained by the concomitant presence of other cardiovascular risk factors. In addition, our multiple regression analysis showed that female gender, hypertension, CAD, hypercholesterolemia, and smoking showed independent correlations with plasma hs-CRP.

## 5. Study Limitations

This study has several limitations. One of which was the cross-sectional design. Although cross-sectional studies can measure association, they are not strong enough to prove causality. It would have been ideal to have obtained serial echocardiograms and hs-CRP levels in our study patients to prove causality. Because our sample size was relatively small, this study may not be representative of the general population or populations from community-based. In addition, our study population consisted of predominantly elderly patients who might have higher hs-CRP levels than younger patients because of aging or nonspecific etiologies. Lastly, we did not assess the severity of MAC. Further study is needed to examine the relationship between systemic biomarkers of inflammation and valvular and/or annular calcification.

## 6. Conclusion

Our study demonstrated that hs-CRP, which is a sensitive marker of systemic inflammation, increased in patients with MAC. This increase in hs-CRP levels in the MAC group persisted in patients without hypertension, coronary artery disease, and in male patients when compared to the control group. There is a need for studies which are designed to present the significance of the relationship of MAC with inflammatory process and its clinical importance.

## Figures and Tables

**Table 1:** Baseline characteristics of the study population.

Variable	MAC Group *n* = 100	Control Group *n* = 100	*P* value
Age, years	65.9 ± 4.8	65.3 ± 4.1	0.11
Women/men (%)	64/36	45/55	0.007
Hypertension (%)	42	28	0.03
Coronary artery disease (%)	37	18	0.003
Diabetes mellitus (%)	26	19	0.23
Hypercholesterolemia (%)	33	24	0.15
Cigarette smoking (%)	17	14	0.55
Asetil salisilic acid use (%)	58	48	0.15
Statin use (%)	42	34	0.24
ACEI or ARB use (%)	34	26	0.21

ACEI: angiotensin-converting enzyme inhibitor; ARB: angiotensin-receptor blocker; MAC: mitral annular calcification.

**Table 2:** Logistic regression analysis for mitral annular calcification.

Variable	Odds ratio	95% CI	*P* value
Age	1.09	1.01–1.17	0.01
Women versus men	2.06	1.11–3.80	0.02
Hypertension	0.62	0.32–1.20	0.16
Coronary artery disease	0.45	0.22–0.90	0.02
Diabetes mellitus	0.79	0.39–1.63	0.53
Hypercholesterolemia	0.63	0.32–1.24	0.18
Cigarette smoking	0.90	0.38–2.01	0.80

CI: confidence interval.

**Table 3:** Hs-CRP levels in the MAC and control group.

	MAC (+)		MAC (−)		*P* value
	*n*	Hs-CRP (mg/dL)	*n*	Hs-CRP (mg/dL)	
All patients	100	2.02 ± 0.35	100	1.43 ± 0.47	<0.001
Females	64	2.05 ± 0.35	45	1.64 ± 0.39	<0.001
Males	36	1.97 ± 0.34	55	1.26 ± 0.47	<0.001
CAD (+)	37	2.10 ± 0.33	18	1.77 ± 0.30	0.001
CAD (−)	63	1.97 ± 0.35	82	1.36 ± 0.47	<0.001
HT (+)	42	1.97 ± 0.39	28	1.90 ± 0.34	0.47
HT (−)	58	2.06 ± 0.32	72	1.25 ± 0.39	<0.001

CAD (+): patients with coronary artery disease.

CAD (−): patients without coronary artery disease, Hs-CRP: high-sensitivity C-reactive protein in mg/dL.

HT (+): patients with hypertension.

HT (−): patients without hypertension.

MAC (+): patients with mitral annular calcification.

MAC (−): patients without mitral annular calcification.

**Table 4:** Multiple linear regression analysis with hs-CRP as the dependent variable.

Variable	*β*	95% CI	*P* value
Age	0.64	−0.006–0.021	0.29
Gender (female)	0.26	0.14–0.39	<0.001
Hypertension	0.19	0.07–0.34	0.002
Coronary artery disease	0.22	0.11–0.40	<0.001
Diabetes mellitus	0.05	−0.08–0.21	0.37
Hypercholesterolemia	0.18	0.07–0.34	0.003
Cigarette smoking	0.15	0.04–0.39	0.016

CI: confidence interval.
